# Mechanisms of vasculogenic mimicry in hypoxic tumor microenvironments

**DOI:** 10.1186/s12943-020-01288-1

**Published:** 2021-01-04

**Authors:** Xiaoxu Wei, Yunhua Chen, Xianjie Jiang, Miao Peng, Yiduo Liu, Yongzhen Mo, Daixi Ren, Yuze Hua, Boyao Yu, Yujuan Zhou, Qianjin Liao, Hui Wang, Bo Xiang, Ming Zhou, Xiaoling Li, Guiyuan Li, Yong Li, Wei Xiong, Zhaoyang Zeng

**Affiliations:** 1 NHC Key Laboratory of Carcinogenesis and Hunan Key Laboratory of Translational Radiation Oncology, Hunan Cancer Hospital and The Affiliated Cancer Hospital, Xiangya School of Medicine, Central South University, Changsha, Hunan China; 2 Key Laboratory of Carcinogenesis and Cancer Invasion of the Chinese Ministry of Education, Cancer Research Institute, Central South University, Changsha, Hunan China; 3 Hunan Key Laboratory of Nonresolving Inflammation and Cancer, Disease Genome Research Center, The Third Xiangya Hospital, Central South University, Changsha, Hunan China; 4 Department of Medicine, Dan L Duncan Comprehensive Cancer Center, Baylor College of Medicine, Houston, TX USA

**Keywords:** Vascular mimicry, Hypoxia, Cancer stem cells, Epithelial-endothelial transition, Extracellular matrix remodeling, Targeted angiogenesis drugs

## Abstract

**Background:**

Vasculogenic mimicry (VM) is a recently discovered angiogenetic process found in many malignant tumors, and is different from the traditional angiogenetic process involving vascular endothelium. It involves the formation of microvascular channels composed of tumor cells; therefore, VM is considered a new model for the formation of new blood vessels in aggressive tumors, and can provide blood supply for tumor growth. Many studies have pointed out that in recent years, some clinical treatments against angiogenesis have not been satisfactory possibly due to the activation of VM. Although the mechanisms underlying VM have not been fully elucidated, increasing research on the soil “microenvironment” for tumor growth suggests that the initial hypoxic environment in solid tumors is inseparable from VM.

**Main body:**

In this review, we describe that the stemness and differentiation potential of cancer stem cells are enhanced under hypoxic microenvironments, through hypoxia-induced epithelial-endothelial transition (EET) and extracellular matrix (ECM) remodeling to form the specific mechanism of vasculogenic mimicry; we also summarized some of the current drugs targeting VM through these processes, suggesting a new reference for the clinical treatment of tumor angiogenesis.

**Conclusion:**

Overall, the use of VM inhibitors in combination with conventional anti-angiogenesis treatments is a promising strategy for improving the effectiveness of targeted angiogenesis treatments; further, considering the importance of hypoxia in tumor invasion and metastasis, drugs targeting the hypoxia signaling pathway seem to achieve good results.

## Introduction

Malignant tumors show rapid growth, poor prognosis, and high mortality; moreover, early diagnosis of such tumors is rather difficult and no effective treatments are available [1]. Tumor cells show continued division and proliferation, consuming large amounts of oxygen and nutrients. When the volume of a solid tumor is less than 2 mm^3^, oxygen and nutrients can be obtained through diffusion. However, upon exceeding this volume, the center of a solid tumor cannot obtain sufficient oxygen and nutrients by means of diffusion alone, and the cells in this region are then exposed to starvation and a hypoxic microenvironment [2, 3]. To meet the demands of continuous proliferation, tumor cells undergo adaptations under the influence of their harsh environment, resulting in progression to a more malignant state. With the gradual growth of tumor tissue, the tumor needs to form new blood vessels to obtain nutrients and oxygen [4]. In recent years, continuous research on angiogenesis in tumors has mostly revealed the pathway of traditional tumor angiogenesis [5]. Based on this model, many targeted drugs have been put into clinical use, but their effect is not very satisfactory. Some researchers have proposed that this may result from the activation of other angiogenesis-related processes [6]. Vasculogenic mimicry (VM) is a recently discovered method of angiogenesis found in many malignant tumors, which provides a new strategy for the clinical treatment of tumor angiogenesis. The vessels formed in VM comprise an arrangement of endothelial tumor cells, supported by periodic acid–Schiff (PAS)-positive cells and rich outer matrix components. These tubes transport nutrients and red blood cells carrying oxygen to the tumor [7]. The mechanism underlying the occurrence of VM has not been fully elucidated, but increasing progress has been made. Research on the soil “microenvironment” for tumor growth suggests that the initial hypoxic environment in solid tumors is inseparable from VM. In the hypoxic tumor microenvironment, some plastic tumor cells such as cancer stem cells (CSCs) demonstrate enhanced stemness and activated differentiation potential. Hypoxia also induces the epithelial-endothelial transition (EET) of CSCs. During this process, CSCs lose some epithelial markers and gain endothelial-like cell characteristics. As the key molecules in EET are almost the same as those in epithelial-mesenchymal transition (EMT), the two processes have often been confused in previous studies. EET has also been described as a subtype of EMT [8]. Considering that this transition is necessary for the VM process, we consider it more appropriate to call it EET; accordingly, this term has been used in the following text to emphasize its differences. This review first introduces the definition of VM in the hypoxic microenvironment and its key links, and then focuses on the molecular mechanisms involving CSCs, EET, and extracellular matrix remodeling in the development of VM in the hypoxic tumor microenvironment.

## Main text

### The key processes of vascular mimicry

Hypoxia is undoubtedly the most typical feature in the microenvironment at the center of a tumor; it is also the most important factor that induces malignant transformation of tumor cells [9] Hypoxia causes a series of changes including metabolic changes, immune escape, angiogenesis, and so on in the tumor. This process is accompanied with the activation of a large number of cellular pathways; of these, hypoxia inducible factor (HIF) is the most critical molecule regulating the expression of a large number of hypoxia-related genes. HIF and its activation mechanism were first discovered and elucidated by Semenza in 1992, who won the 2019 Nobel Prize in Physiology or Medicine along with Ratcliffe and Kaelin for their outstanding contributions to the oxygen sensing pathway [10, 11]. In the hypoxic microenvironment, tumor cells form new blood vessels to obtain the required oxygen and nutrients for supporting their continued proliferation. A large number of studies have shown that hypoxia is closely related to the development of VM. For instance in a mouse model of melanoma, mice in the ischemic model group were found to demonstrate increased VM compared to the control group, which was positively correlated with HIF-1α and HIF-2α expression, indicating that oxygen deficiency promoted VM [12]. Further, a large number of studies have shown that hypoxia or HIF molecules promote the VM process in multiple tumor types including cervical cancer [13], small cell lung cancer [14], liver cancer [15], and gallbladder cancer [16]. HIF-1 can also directly regulate the expression of multiple VM-related molecules such as VEGF, Twist, LOX, MMP2, and MIFect [17].

In 1999, Maniotis et al. discovered a tube-like structure composed of melanoma cells differentiated into cells with endothelial-like characteristics in malignant melanoma. No vascular endothelial cells were found beside these ducts. However, these ducts were found to contain red blood cells and other substances, suggesting that their role was the same as that of traditional blood vessels. Therefore, the formation of such structures is referred as VM [7]. VM phenomenon has been found in cancer such as hepatoma, ovarian cancer, gastric cancer, prostate cancer, and nasopharyngeal cancer, and a large number of clinical data show that VM is closely related to high tumor aggressiveness and poor patient prognosis [18, 19] Tumor cells demonstrating VM also have very aggressive and metastatic characteristics including tumor cell markers, endothelial cell markers, plasticity/stemness markers, and so on [20]. VM structures are described as high perfusion, matrix-rich tubular, or pattern-like matrix structures containing collagen, heparan sulfate proteoglycans, and plasma. Unlike traditional angiogenesis, vascular endothelial cells and fibroblasts do not show VM, which is characterized by the dense deposition of tumor cells and extracellular matrix. The glycoproteins forming these structures include type I, IV, and VI collagen, and laminin Ln5 and its cleavage products, γ2x and γ2´ [7, 21]. Presence of CD31/CD34-negative and PAS-positive cells as well as red blood cells in the tube are generally used as the identification criteria for VM [22, 23]. However, some studies have recently indicated that the CD31−/PAS+ marking method is not accurate, and that it is necessary to find a more suitable method [24]. In glioblastoma, an arrangement of CD34-negative cells was found in a vascular tube containing red blood cells, and these cells were tumor cells. Subsequently, serial tissue sectioning and staining were performed to determine the nature of the duct. A region of connection between the CD34-negative and positive cells was found, indicating that the duct structure was continuous with normal blood vessels [25].

**Fig. 1:**
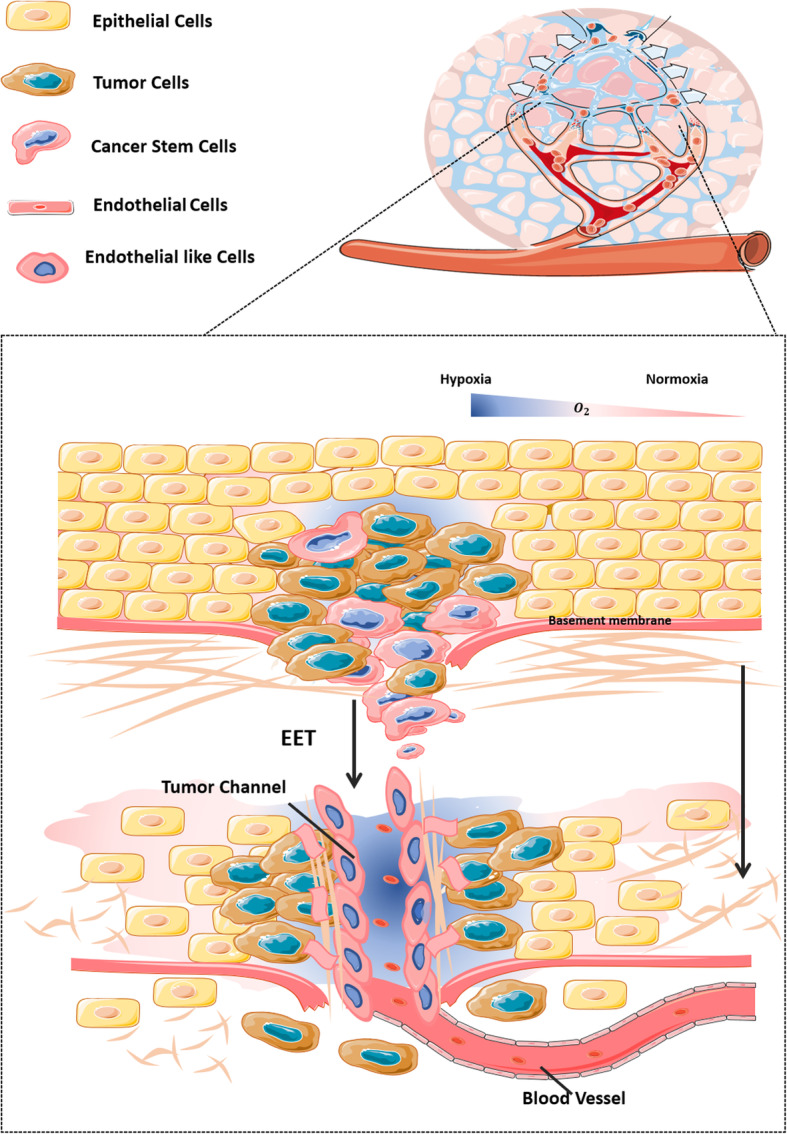
The key processes involved in vasculogenic mimicry under hypoxia

Cells that have undergone VM are directly exposed to the blood stream, and the shed cells are easily transferred with blood. Therefore, VM generally occurs in highly invasive, highly metastatic, and advanced malignant tumors, often indicating poor patient prognosis [26]. Although the specific mechanism underlying the induction of VM has not yet been fully elucidated, we consider that the process involves the transformation of tumor parenchymal cells and changes in the tumor extracellular matrix components. The specific mechanisms include CSCs, EET, and ECM remodeling (Fig. 1).

HIF enhances the stemness and differentiation potential of cells with strong plasticity in the highly malignant tumor cell population (especially tumor stem cells) in the tumor microenvironment; these tumor cells then transform to more mobile cells through the EET process induced by hypoxia. Then, with cell stretching and lengthening, the expression of related transcription factors, Twist and Snail in the cell is upregulated, causing downregulation of the tight junction protein, E-cadherin, between epithelial cells, along with upregulation of molecules related to angiogenesis such as VE-cadherin and fibronectin. These cancer stem cells then demonstrate the characteristics of endothelial cells [27]. Through a series of intracellular signaling pathways, highly malignant tumor cells express high levels of matrix metalloproteinase and other substances, degrading some substances such as laminin and remodeling the ECM, which promotes the migration of tumor cells and their transformation to a malignant phenotype, providing a stretching space and soil for the formation of a VM network [9, 28]. These upregulated proteins then arrange tumor cells through duct-like adhesions to form duct-like structures together with the remodeled extracellular matrix; finally, these ducts infiltrate and extend into the vascular network, and begin to transport red blood cells and nutrition to the tumor cells.

### Hypoxia promotes cancer stem cell differentiation into an endothelial-like phenotype

The American Cancer Society defines cancer stem cell-like cells (CSCs) as a tumor cell type with self-renewal ability and multiple differentiation potential [29]; these form only a small part of tumor cells. Increasing researchers have indicated that differentiated tumor cells can regain stemness and become tumor stem cells through a process involving multiple mechanisms, including the indispensable effect of hypoxia on CSCs [30]. Experiments have proved that after serial cloning in vitro and transplantation in vivo, globular cells with pluripotent melanoma still exist, renew themselves, and form new tumors [31]. CSCs can also differentiate into various cells. For instance, glioblastoma stem cell-like cells (GSC) cultured in vitro can produce cells with an endothelial phenotype and functional characteristics under conditions conducive to endothelial cell differentiation [32, 33].

Currently, CSCs are known to directly participate in the development of VM in triple negative breast cancer [34] and melanoma [35]. CSCs can be effectively located using surface markers such as ATP binding cassette transporter, aldehyde dehydrogenase (ALDH), CD133, CD44, and so on [36–38]. These markers of CSCs also show a significantly positive correlation with tumor invasion, metastasis, and poor prognosis. In three-dimensional cell cultures, VM cells of melanoma expressing CD144 (VE-cadherin) have been found to express the CSC marker CD133; if this stem cell marker is silenced, the ability of the tumor to form a VM network is reduced significantly [35]. Further, in triple-negative breast cancer, ALDH1+ tumors and CD133+ tumors express more VE-cadherin and Twist1 compared to ALDH1(−) tumors and CD133(−) tumors, and demonstrate the ability to form VM-like channels.

However, the mechanism by which CSCs differentiate in a direction similar to endothelial cells is unclear. In an in vitro study, pretreatment with collagen matrix could induce invasive melanoma cells capable of forming VM; however, the generation of invasive melanoma cells was significantly less than that induced by the in vivo microenvironment [39]. CSCs injected into mice together with stromal cells extracted from the tumor environment are known to form more aggressive tumors, indicating that the matrix surrounding the CSCs plays an important role in tumor progression [40]. These observations indicate that the microenvironment significantly impacts the potential of CSC to differentiate into different cell phenotypes. This interaction has an indispensable influence on the development of VM. Therefore, we focus on the maintenance of CSC stemness in the microenvironment and how the microenvironment promotes CSC transformation into incomplete endothelial cells that demonstrate VM. We believe that CSCs are located in a small and special tumor microenvironment sub-chamber niche, called the CSC niche. This niche mainly contains cellular and non-cellular components similar to the tumor microenvironment, including fibroblasts (CAFs), endothelial cells (ECs), immune cells, mesenchymal stem cells (MSCs), tumor-associated macrophages (TAMs), and ECM [41].

**Fig. 2:**
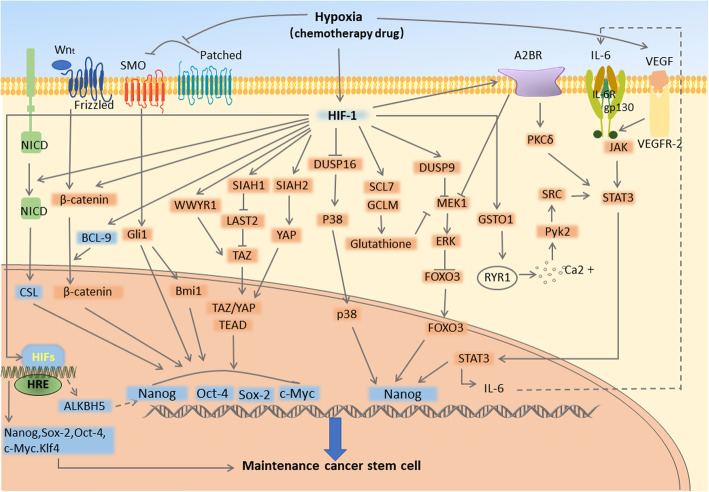
The main pathways by which hypoxia promotes CSC stemness and differentiation potential

Hypoxia is also an important regulatory factor in the CSC niche; hypoxia promotes CSC transformation to endothelial cell-like structures from many aspects. Increasing studies on hypoxia and HIF signaling pathways in recent years have shown that the hypoxic microenvironment plays an important role in regulating the phenotype and function of CSCs in liver cancer [42], cholangiocarcinoma [43], colorectal cancer [44], and breast cancer [45]. It may indirectly or directly act on related transcription factors such as c-Myc, Sox-2, and Oct-4 by inducing CSC markers, adenosine/STAT3/IL-6 pathway, MAPK/ERK pathway, Notch, Wnt, Hedgehog, Hippo signaling pathway, and so on, to promote the multi-directional differentiation potential of CSCs [46] (Fig. 2).

Under hypoxic conditions, the expression of CSC-specific surface markers is also upregulated [47, 48]. Among them, CD133 is a cell surface glycoprotein. Molecular analysis of the CD133 promoter shows that HIF-1α and HIF-2α can form a complex with ELK1 to bind the CD133 promoter sequence. Simultaneously, hypoxic conditions induce significantly increased expression of IL-6, which can activate and stimulate the rapid transfer of STAT3 to the nucleus, and its binding with the promoter region to increase the protein expression of CD133. Active STAT3 can also recruit the HIF-1α promoter to stimulate CD133 expression through HIF-1α/IL-6/STAT3 [49]. CD44 expression is also dependent on HIF-2α and promotes the growth and stem cell-like phenotype in glioma [50]. These stem cell surface markers are also found to be involved in the induction of VM in different cancer types [51–54].

Hypoxia can also maintain the stem-like phenotype of cancer stem cells by activating the expression of stem cell-related factors such as c-Myc, Sox-2, and Oct-4, and contributing to VM [43, 55]. Oct4, Sox-2, Klf4, Nanog, and c-Myc are other stem-related transcription factors that can act as reprogramming factors, called OSKM Yamanaka factors or Y4. High expression of these factors can be recognized as functional markers of CSCs in tumors and can be used to control the CSCs. MYC, an important regulator of pluripotency, self-renewal, and maintenance [56], can bind to Nanog and Sox-2 simultaneously by differentially binding to the HIF-2α promoter [57]. Oct-4 and Sox-2 are the most important and most researched protein regulators of Nanog. They form a complex with Klf4 and bind to the Nanog promoter to induce its transcription [58]. HIF can increase Nanog levels in cancer cells by controlling the stability of Nanog mRNA. HIF-dependent ALKBH5 can demethylate Nanog; Nanog mRNA further shows increased stability after demethylation and induces an increase in the stemness factor expression of CSCs [59].

In a hypoxic environment, activation of some pathways can indirectly affect the expression of pluripotent stem cells [60]. Among them, the Notch signaling pathway activated by HIF-1α under hypoxic conditions, is essential for maintaining the stemness of cancer stem cells [61]. The Notch receptor is cleaved by γ-secretase into a stable intracellular domain (NICD), which is released from the membrane and transferred to the nucleus, where it interacts with members of the CSL DNA-binding protein family and induces transcriptional changes [62]. HIF-1 can bind directly to the Notch promoter to enhance the expression of its downstream target gene, Notch [63]; further, HIF-1α accumulated in the cytoplasm under hypoxic conditions can directly interact with the intracellular structure of Notch. The NICD interacts with Notch facilitating its nuclear translocation after being cleaved [64]. Activation of the HIF-1α and Notch pathways in ovarian cancer can directly stimulate Sox-2 promoter activity [65]. HIF-1α can also form a transcription complex with STAT3, and target the gene Vasorin, which can effectively stabilize Notch1 protein on the cell membrane to form NICD1, and play a role in preventing Numb (an adaptor protein that inhibits Notch signaling)-mediated lysosomal degradation [66]. Notably, HIF-2α exhibits the opposite inhibitory effect after binding to NICD, and competes with HIF-1α for binding NICD in glioblastoma [67]. Further, crosstalk with the Wnt channel may also determine the overall effect of the Notch signal [68].

Wnt/β-catenin is also an important signaling pathway in the regulation of CSC self-renewal [69–71]. In hypoxia, the Wnt signal is turned on, and the cell surface receptors (LRP-5/6 and Frizzled family proteins) bind to the secreted Wnt ligand. Stable β-catenin translocates to the nucleus, interacts with the LEF/TCF family of transcriptional activators, and then binds and activates the downstream and CSC-related target gene sites such as c-Myc, Oct-4, Sox-2, and Nanog [72]. HIF-1α functions both upstream and downstream of the Wnt/β-catenin signaling pathway, and these pathways have mutual regulation effects [73]. Under hypoxic conditions, HIF-1α can induce the expression of BCL9, an indispensable co-activator that effectively enhances β-catenin-mediated transcriptional activity [74]. The β-catenin co-transcription factors, LEF and TCF, in the nucleus, can also be directly regulated by HIF-1 molecules [75, 76]. Similarly, HIF-2α can increase the expression of Y4 indirectly by activating the Wnt pathway to promote the stem cell characteristics of tumor cells [77]. Oct-4 and c-Myc overexpression can increase the stemness of CSCs and promote VM production, which significantly increases tumor invasion and metastasis [78, 79]. Notably, in a study of defective renal clear cell carcinoma, HIF-1α inhibits the transcriptional activity of c-Myc by directly blocking the interaction between c-Myc and its DNA binding partners (Max, Sp1, Miz1). HIF-2α promotes c-Myc activity by recruiting these binding partners or directly forming a complex with them [80].

Hedgehog signaling also contributes to maintain the CSC stemness phenotype during hypoxia. Hedgehog signal activation is controlled by two receptors, Patched (12-pass transmembrane receptors, Ptch1 and Ptch2) and Smo. Patched receptors inhibit Hedgehog pathway activation, whereas Smo receptors have the opposite effect [81]. Suppression of Smo by Patched is reduced under a hypoxic environment. By binding to the Hh family ligand SHH, Hh targeting genes can be activated. The Hh pathway component Gli1 can be transported to the nucleus to induce the expression of its downstream stemness transcription factors, Nanog, Oct-4, and Sox-2; Gli1 can also bind to the Bmi1 promoter to increase Bmi1 transcription [82], thereby jointly enhancing the tumor stem phenotype in a hypoxic environment [83].

HIF-1α can also promote CSC self-renewal and tumor initiation ability by triggering the expression and activity of the Hippo pathway effector, TAZ (transcriptional coactivator with PDZ binding motif), and its related protein, YAP (Yes-related protein) [84]. Under hypoxic conditions, HIF-1 activates the effector TAZ of the Hippo pathway through two separate mechanisms: first, it directly binds to the WWTR1 promoter to increase its expression, and then forms a complex with WWTR1 to promote TAZ transcription; second it promotes the transcription of SIAH1/SLAH2, which induces ubiquitination of LATS2 (a kinase that inhibits TAZ nuclear localization) [85, 86]. The subsequently activated TAZ/YAP forms a complex with TEAD to drive downstream target transcription [87]. A TEAD binding site has been found on the pluripotency factor Sox-2. YAP1 can induce Sox-2 mRNA transcription through interaction of the WW domain with the Oct-4 transcription factor. If YAP1 is depleted or inhibited by siRNA, the angiogenic network-forming ability of CSCs is weakened [88].

A hypoxic environment also protects CSCs from chemotherapy drugs through acquisition of a stem phenotype. In triple-negative breast cancer, chemotherapy drugs such as paclitaxel or gemcitabine can induce HIF to increase the multidirectional differentiation potential of cancer stem cells [89]. HIF-dependent negative regulators of MAPK such as DUSP9 and DUSP16, whose activation and inactivation can respectively trigger ERK inactivation and p38 activation, can increase the expression of pluripotency factors in various ways. For instance, ERK inactivation leads to FOXO3 dephosphorylation and activation, which can activate Nanog transcription [90]. Activation of p38 results in the phosphorylation and inactivation of ZFP36L1 (zinc finger protein 36 C3H type 1), leading to the stability of Nanog and Klf4 mRNA [91]. Activation of HIF-dependent SLC7A11 (cystine transporter xCT) and GCLM (glutamate-cysteine ligase) under induction by chemical drugs can increase the synthesis of glutathione, which can interact with MEK1 kinase. Chelation of the cofactor copper blocks the MEK1-ERK signaling pathway, leading to FOXO3 nuclear translocation and transcriptional activation of the gene encoding the pluripotency factor Nanog [90]. HIF-dependent GSTO1 (glutathione transferase) can increase the Ca^2+^ level in the cytoplasm, thereby activating the PyK2/SRC pathway, and enabling STAT3 activation and nuclear translocation [92]. HIF-1α-dependent adenosine receptors can also increase STAT3 phosphorylation through Ca^2+^-independent PKCδ (protein kinase C), thereby promoting STAT3 nuclear translocation. STAT3 activates the transcription of Nanog, and also binds the IL-6 promoter to generate a positive feedback loop. IL-6 then activates JAK2 (tyrosine kinase) and STAT3 (at Y705, by binding its homologous receptor) phosphorylation [93], and enhances the expression of stem cell factors in a positive feedback loop [94]. HIF-1-dependent adenosine receptors have also been speculated to inhibit the ERK signaling pathway, leading to FOXO3 nuclear translocation and transcriptional activation of genes encoding the pluripotency factor Nanog. As activation of the PI3K/mTOR/mTOR signaling pathway is very common in patients with breast cancer [95], and western blot analysis has revealed that the main signaling molecules of this pathway are positively correlated with HIF-2α expression, HIF-2α may also maintain the stemness of human breast cancer CSCs through the PI3K/AKT/mTOR signaling pathway [96].

VEGF, also called VEGF-A, is also an important factor in CSCs and VM. Increased VEGF-A is significantly related to the development of VM in salivary adenoid cystic carcinoma [53] and esophageal cancer [97]. VEGFR-2 is the most important receptor of VEGF, and can form a complex with NRP (a neurofibrin) to enhance its affinity for VEGF [98]. These molecules are also closely related to the self-renewal, proliferation, and differentiation of cancer stem cells [99–101]. A hypoxic environment can activate VEGF [53], which binds to its receptor VEGFR-2 through an autocrine or paracrine mode, and activates JAK2/STAT3 to stimulate the stem transcription factors c-Myc and Sox-2. Thus, they can regulate CSC differentiation, survival, proliferation, and migration [102, 103]. VEGF expression in melanoma is reported to promote the development of VM through activation of the PI3K/AKT pathway in the tumor microenvironment [104].

### Hypoxia promotes vasculogenic mimicry by facilitating epithelial to endothelial transition

**Fig. 3:**
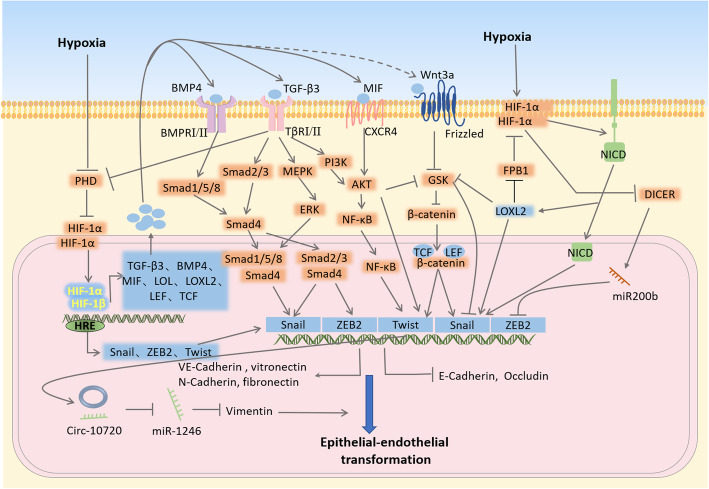
The main pathways through which hypoxia promotes EET to induce VM

EET is the biological process of epithelial cells transforming into cells with an endothelial phenotype through specific procedures. The relationship between EET and EMT has been described above; however, the process of EET is driven by hypoxia as many studies have shown that some molecules and signal pathways related to EET are indirectly or directly affected by hypoxia [105] (Fig. 3).

During EET, tumor cells lose their polarity and tight junctions, and gain the ability to infiltrate and migrate. This process is accompanied by the transition from epithelial marker molecules to endothelial marker molecules, such as decreased expression of tight junction proteins such as E-cadherin and occludin, and increased expression of vimentin, VE-cadherin (adhesion molecule of endothelial cells), fibronectin, vitronectin, and other molecules [106, 107]. Among these, E-cadherin is a tight junction protein between epithelial cells, and its expression is significantly downregulated in VM [108]. VE-cadherin plays an extremely important role in VM [109]. In liver cancer, Sun et al. studied Twist, a key molecule of EET, and the development of VM, and they revealed a key relation between EET and VM contact [110]. Additionally, many related molecules in the EET process can promote VM [27]. Increasing studies have found that in epithelial ovarian cancer [111], colorectal cancer [112], kidney cancer [113], liver cancer [114], salivary gland cancer [53], and other malignant tumors, HIF-1α induces VM by inducing EET. Under hypoxic conditions, this process is influenced by the activation of TGF-β [63], Notch [115], and the Wnt/β-catenin pathway [116], as well as the interaction and cross-linking of EET-related transcription factors (such as Twist, Snail) [117].

Let us first describe how the transcription factors Twist, Snail, and ZEB2 affect the EET and VM processes. First, these transcription factors are directly regulated by hypoxia. HIF-1 can bind to the hypoxia response element (HRE) on the Twist, Snail, and ZEB2 promoter sequences in the nucleus to play a regulatory role [118–120]. Twist protein, a helix-loop-helix transcription factor, belonging to the bHLH transcription factor family, is encoded by the Twist gene located on human chromosome 7p21, and contains two transcription factors, Twist1 and Twist2 [107]. TWIST expression can downregulate the expression of multiple epithelial genes and activate the expression of endothelial genes [106]. Twist1 can use an accessory protein to open the nuclear membrane pores, enter the nucleus, and participate in the transcriptional regulation of a variety of downstream genes. Twist1 overexpression can induce tumor cell metastasis, whereas Twist2 is involved in the tumor growth stage; these two proteins have different functions in different tumor types [121] The bHLH domain can specifically bind to the E-box domain on DNA to play a regulatory role [122].

Through the above-mentioned binding regulation, Twist1 can reduce the expression of E-cadherin and upregulate VE-cadherin [123, 124]. In breast cancer, Twist1 overexpression can significantly inhibit E-cadherin transcription [125]. Twist2 also has a similar effect. In pancreatic cancer cell lines cultured under hypoxic conditions, E-cadherin expression was inhibited by Twist2 binding to the E-box domain; notably, although Twist1 expression was also increased under hypoxia in this model, Twist1 did not show the ability to bind E-cadherin. This suggests that the specific regulatory mechanisms of different cancer models are different [126]. Vimentin constitutes an important component of the cytoskeletal intermediate filament, and is upregulated during VM. Hypoxia-induced vimentin overexpression is known to promote VM in renal cell carcinoma cells [113]. In hepatoma, Twist1 can promote vimentin expression, and it has been verified that Twist1 is combined with a circular RNA, the promoter region of CUL2 (Cullin-2), and promotes the mRNA of vimentin by adsorbing the microRNA targeting vimentin degradation, resulting in EET [127]. In addition, Twist1 can also affect VEGFR2 expression; for instance, VEGFR2 expression was significantly reduced in the eyes of mice with Twist1 knocked out [128]. Twist1 has a very complex mechanism of action, including interaction with other molecules. For example, under hypoxic conditions, Twist1 has a direct regulatory effect on Bmi1, a member of polycomb-complex 1 (PRC1). After Twist1 activates Bmi1, they form a complex that acts on the E-box area of E-cadherin. The complex formed by these two molecules can effectively inhibit its expression and promote EET and VM [114, 129]. Later studies have proved the above mechanism in hepatoma, and shown that these two molecules can increase tumor cell stemness and promote VM [18]. The Bcl-2 family inhibits apoptosis induced by various stimuli (such as hypoxia). Bcl-2 itself can promote the occurrence of hypoxia-induced VM by promoting VE-cadherin expression [130]. Bcl-2 contributes to the stability and intranuclear localization of Twist [131]. In gastric cancer, MACC1 promotes Twist1/Twist2 expression through HGF/cMet and promotes VM [132]. Twist is also related to the signaling pathways mentioned below. Twist functions as a transcription factor to promote ROR1 (wnt5a receptor) transcription, and promotes EET through the non-classical Wnt pathway [133].

Snail, a member of the zinc finger protein superfamily has a unique zinc finger structure [134]. Similar to Twist, Snail can promote VM by inducing EET. High expression of the Snail family is important for the development of VM. For example, overexpression of Slug, a Snail family member, can maintain the CSC phenotype and promote VM [135]. All Snail family members have a highly conserved carboxy-terminal binding region, which can recognize and bind the E-box region of the target gene, thereby inhibiting E-cadherin and promoting VE-cadherin expression to promote EET [136]. Some studies have also proved that a decrease in VHL (the molecule that degrades HIF-1 by ubiquitination) causes an increase in Snail and SIP1, which leads to EET [137]. Snail also interacts with other molecules. For instance, Snail is dually regulated by GSK-3β. GSK-3β has two motifs that can bind to Snail, one of which is phosphorylated to inhibit degradation, and the other binds to Snail and facilitates its nuclear transfer [138]. Further, LOX/LOXL2, a family of lysyl oxidases, catalyzes the covalent cross-linking of collagen and elastin in ECM [139]. LOXL2 upregulation decreases E-cadherin, increases vimentin, and promotes the process of EET and VM [15, 140, 141]. Its acts by interacting with GSK in the cytoplasm to stabilize the expression of Snail protein and ultimately increases its nuclear storage [142]. LOXL2 is regulated by hypoxia and can promote the hypoxia pathway. In liver cancer, HIF-1α promotes LOXL2 expression, which promotes EET and VM through the Snail/FBP1/VEGF pathway [143]. The tumor suppressor protein p53-induced nuclear protein 1 (TP53INP1) plays an inhibitory role in tumor invasion and metastasis and has been shown to inhibit hypoxia-induced EET through the ROS/GSK-3beta/Snail pathway [144].

ZEB family transcription factors, ZEB1 and ZEB2, bind to regulatory gene sequences on the E-box, and inhibit or activate the effects of target genes. ZEB often needs to recruit the C-terminal binding protein (CTBP) to perform its transcriptional repressive function. ZEB overexpression is also related to the development of VM. ZEB promotes VM by promoting the EET process [145].

Multiple signaling pathways related to hypoxia affect the expression of these transcription factors. NF-κB can upregulate the Twist expression and promote the EET process [146]. Recent studies have found that NF-κB/p65 can bind to the promoter regions of EET-related transcription factors such as TWIST1, SLUG, and SIP1 to promote their transcription [147]. Notably, the relationship between the NF-κB signaling pathway and Twist is complex, and involves a loop of negative feedback regulation [148]. The NF-κB pathway is activated under hypoxic conditions due to increased degradation of the NF-κB inhibitor, IκBα, resulting in downstream reactions. Although HIF-1 expression is related to NF-κB activation, its specific mechanism still needs to be explored. NF-κB can also be activated by the PI3K/AKT pathway, which is affected by hypoxia [149]. The Wnt pathway also influences EET [150, 151]. For example, Wnt3a enhances the expression of VEGFR2 and VE-cadherin through Wnt /β-catenin signal activation, thereby promoting VM [152]. Other studies have shown that Wnt3a does not promote EET under normoxia, but induces EET under hypoxia in a HIF-1a-dependent manner, and promotes VM [116]. The Notch pathway can also affect and promote VM [153, 154]. Upon Notch pathway activation, Snail expression is promoted in two ways. After HIF-1α cleavage, the NICD is released from the cell membrane into the nucleus, and can directly bind to the promoter of Snail to promote its transcription. Further, translocation of NICD into the nucleus can also increase the binding of HIF-1 and LOX, thus promoting the transcription of LOX, which can stabilize the Snail protein [155].

TGF-β is a cytokine discovered in the 1980s [156]. The TGF-B signaling pathway mainly participates in the EET process through Smad-dependent as well as Smad-independent pathways [157]. Smad protein is the main signal molecule for intracellular transduction in the TGF-β Smad-dependent pathway. Smad protein is present in the cytoplasm and can transmit signals from the cell membrane to the nucleus, thereby regulating gene transcription [158]. TGF-β acts on cell surface receptors, followed by the phosphorylation of type I receptors by type II receptor kinase; this phosphorylation of receptor complexes induced by TGF-β activates Smad2 and Smad3. Subsequently phosphorylated Smad2 and Smad3 form trimers with Smad4, which are transferred to the nucleus, where they combine with DNA-binding transcription factors and act in concert to activate or inhibit the transcription of target genes [159, 160]. These target genes include EET-related transcription factors Snail, ZEB, and so on [161]. TGF-β can also regulate Snail expression through a Smad-independent pathway, which is cross-linked with the Wnt pathway. The TGF-β receptor can interact with the regulatory region of PI3K, causing activation of the PI3K/AKT pathway, inhibiting GSK, increasing the amount of β-catenin, and directly promoting Snail expression [162, 163]. TGF-β can activate downstream extracellular regulatory protein kinases through the mitogen activated protein kinase (MAPK) pathway, affecting the translation of target genes, and simultaneously enhancing the transcription enhancing activity of the Smad-dependent TGF-β pathway [164, 165]. The above pathways all promote EET-related transcription and translation, as well as tight junction formation in a Smad-independent manner through TGF-β. BMP4, a member of the TGF-β superfamily, is regulated by HIF-1α [166]. After binding the related membrane receptors, BMP4 activates its downstream genes through Smad-related signaling pathways. Further, it can upregulate the EphA2/VE-cadherin/MMP2 signaling pathway, and simultaneously promote the maintenance of stemness in stem cells through EMT, and promote VM network formation in liver cancer [167]. Related studies have reported that activation of the classical BMP signaling pathway, Smad1/5/8 is necessary in Snail-induced EMT [168]. In addition, Nodal, another important molecule of the TGF-β superfamily, upregulates Slug through the Smad2/3 pathway, and Snail and c-Myc induce EMT, thereby promoting VM [169]. The TGF-β pathway is also regulated by hypoxia. In some cancer cell types, TGF-β3 levels are directly regulated by HIF-1 [170]. However, this is not true for all cancer cell types. For instance, in hepatocellular carcinoma, hypoxia does not increase the expression of TGF-β, but increases the protein content of its active forms. HIFs regulate TGF-β, and the amount of related activating protein activates the TGF-β pathway [171]. Interestingly, there is a two-way regulatory relationship between TGF-β and HIF-1 molecules. TGF-β can reduce the expression of PHD, the upstream inhibitory molecule of HIF-1α, through Smad-independent pathways, thereby promoting HIF-1α accumulation [172].

An increase in HIF-1α expression under hypoxic conditions can promote binding between the DICER enzyme and Parkin, cause ubiquitination and degradation of DICER, ultimately reduce the level of intracellular DICER, and reduce the inhibition of ZEB transcription factors [173]. MIF (macrophage migration inhibitory factor) is a target gene of HIF-1 [174]. MIF production is greatly increased under hypoxic conditions, which activates the CXCR4 receptor on the cell membrane and activates the AKT pathway to induce EET-related transcription factors.

### Hypoxia promotes vasculogenic mimicry network formation by promoting extracellular matrix remodeling

**Fig. 4:**
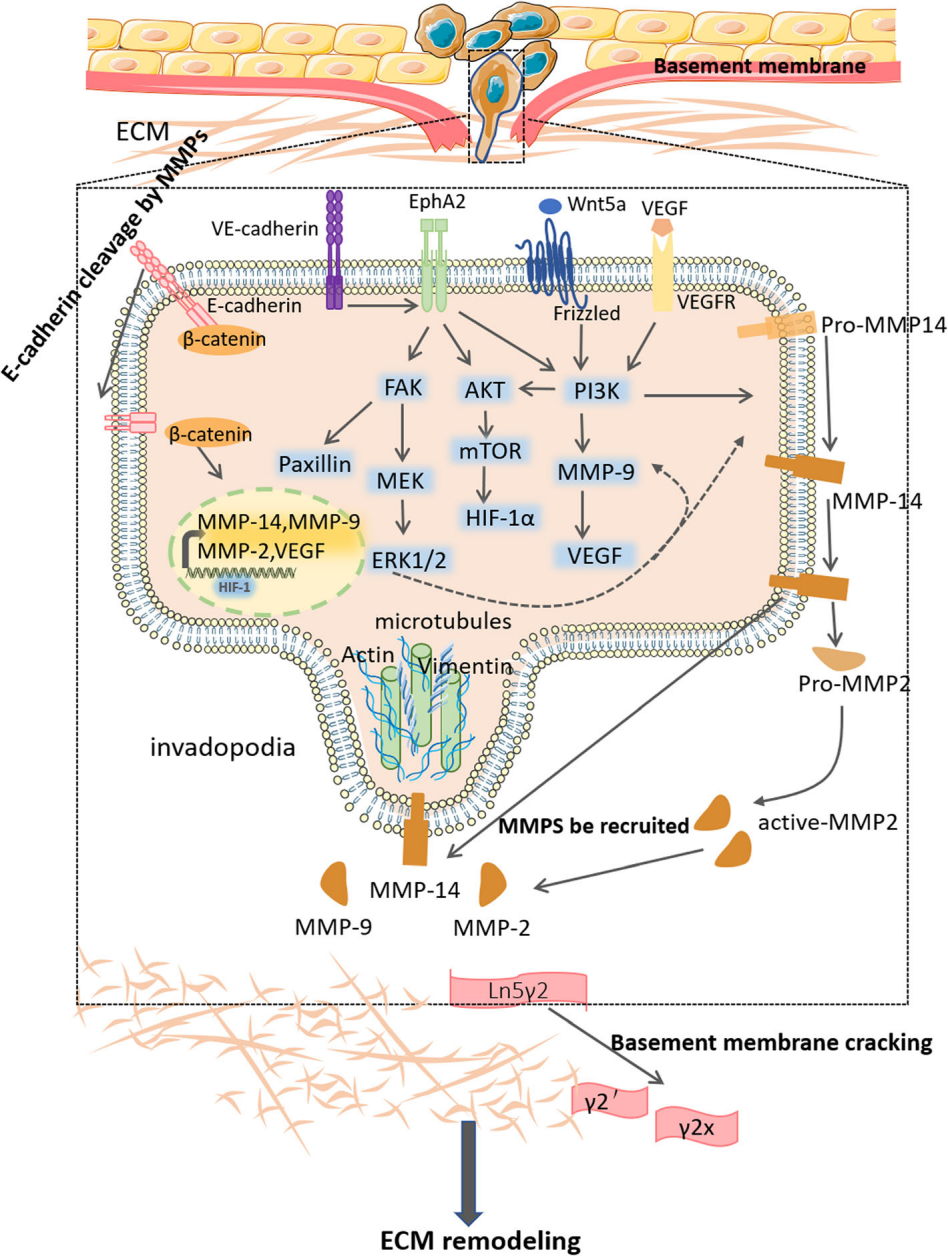
The main mechanisms by which extracellular matrix remodeling promotes the occurrence of VM

Under the influence of the hypoxic microenvironment, reshaping the extracellular matrix (ECM) is very important as cancer cells produce a lot of MMPs (matrix metalloproteinases) for ECM degradation; this can induce cell biological responses such as activation of signaling pathways, simultaneously providing space for the formation of VM blood vessels and reducing resistance to cell morphological changes during VM. Changes in ECM components can activate nuclear signaling pathways associated with CSCs and EET. In addition, cells in the ECM can also secrete type IV collagen, PAS-positive substances, and other extracellular matrix components to participate in the formation of basement membrane-like substances that line the inner wall of VM networks (Fig. 4).

MMPs are particularly important in the process of ECM degradation. Under hypoxic conditions, high expression of MMP molecules increases tumor invasiveness and promotes VM [175]. Production of MMPs is also related to the EET process as activation of ZEB2 [145], Snail [176], and TGF-β [175] signaling pathways as mentioned above, can all promote MMP production. MMPs are directly regulated by the hypoxia inducible factor HIF-1 as MMP-2, MMP-9, and MMP-14 are all its target genes. Thus, the ECM degradation process is also closely related to hypoxia [17].

MMP-2 and MMP-9 can both degrade ECM components and the basement membrane. They can promote the formation of VM blood vessels and increase the invasiveness of tumor cells under hypoxia [12, 177]. In addition, MMP9 can promote the release of VEGF and induce angiogenesis [178]. MMP-2 and MMP-9 can release the precursor TGF-β stored in ECM in a CD44-dependent manner [179]. In the early stage of VM formation, MMP also lyses cell ECM adhesion proteins and cell connexins and releases single epithelial cells from the epithelial layer; certain lysed fragments then initiate signal transduction pathways from the outside to inside, leading to extensive changes in gene transcription patterns. MMP-3 and MMP-9 can both cleave E-cadherin [180, 181]. Under ischemic conditions, MMP-14 is indispensable for E-cadherin cleavage. This results in release of β-catenin bound to E-cadherin from the cell membrane, and its degradation or nuclear translocation to activate the transcription of nuclear targets through the classical Wnt pathway; this indicates cross-links between the pathway and EETs [182, 183].

MMP-14 is a membrane-type matrix metalloproteinase mainly located on the cell membrane. In addition to degrading gelatin itself, MMP-14 can also activate pre-MMP-2 and convert it to an active form of MMP-2. MMP-2 exerts a “drill” effect causing the lysis of Ln5γ2 (laminin) and finally promotes the formation of VM ducts; this is considered the classic reaction of VM duct formation [184]. This pathway is regulated by multiple other molecules. Endothelial cadherin (VE-cadherin), also known as cadherin 5 or CD144, is a cell adhesion protein usually expressed in endothelial cells [109, 185]. In mice transplanted with small cell lung cancer cells, the VM network-forming cells are identified as xenogeneic tumor cells that highly express VE-cadherin, which is essential for tumor aggressiveness and VM, and is involved in the adhesion between endothelial cells; VE-cadherin expression is significantly increased under hypoxia [186, 187]. EphA2 is a transmembrane tyrosine kinase receptor in human epithelial cells and its high expression in prostate cancer cells is found to be significantly related to the poor prognosis of patients [188, 189]. VE-cadherin can induce EphA2 to relocate to the cell membrane in VM, and can cause EphA2 phosphorylation [190]. EphA2 activates the PI3K/AKT pathway by activating PI3K. On one hand, EphA2 can increase the expression of MMP9. On the other hand, it can convert pre-MMP14 into activated MMP14, thereby activating the classical pathway [191, 192]. PKA is also involved in this process as a negative regulator. Hypoxia activates PKA, which inhibits the MEK/ERK pathway and downregulates the expression of MMP14, MMP2, and others [193]. Under hypoxic conditions, VEGF, whose expression is greatly increased, can also participate in ECM remodeling by activating the PI3K/AKT pathway [104]. Wnt5a is reported to activate PI3K through the non-canonical Wnt5a/Ca^2+^ pathway, regulate the above pathways, and affect VM development in ovarian cancer [194]. In addition, EphA2 can also activate FAK in melanoma. On one hand, FAK mediates the phosphorylation of Paxillin at local adhesion sites, thereby regulating focal adhesion, increasing tumor cell fluidity, and contributing to VM development [195]; on the other hand, FAK regulates MMP-2 and MT1-MMP activities through Erk1/2, thereby promoting ECM remodeling [196]. Lysed components of Ln5 γ2’ and γ2x fragments promote cell migration and VM. Studies have shown that only highly aggressive melanomas express Ln5 and its fragments [197]. MMPs and γ2’ and γ2x fragments can synergistically form migration signals and pre-migration fragments. These fragments are involved in inducing an aggressive angiogenic phenotype in poorly invasive tumor cells, which is critical in for VM [9]. In addition to transmitting signals to induce cells, γ2’ and γ2x fragments are also the main materials that constitute the PAS+ supporting components in the walls of VM tubes, which can regulate the assembly of endothelial cells into a tubular structure [198].

The EMT transcription factor Twist is reported to induce the formation of actin-enriched membrane protrusions called aggressive pseudopods (invasive pseudopods are a special actin-based membrane protrusion found in cancer cells, and degrade ECM by targeting proteases); aggressive pseudopodia can recruit MMP-2, MMP-9, and MMP-14 to the leading edge where they degrade collagen and the basement membrane ECM, thereby promoting tumor invasion and metastasis [199, 200]. These types of invasive pseudopodia are more common in cells demonstrating VM, but there are few in-depth studies on their specific regulatory mechanisms [201]. The abundant matrix components involved in VM, including collagen І, IV, and VI, are also involved in VM development. In vitro experiments have shown that pre-treated collagen matrix can induce poorly invasive melanoma cells to express key angiogenesis/matrix remodeling related genes for the first time [202]. Collagen IV is a component of the basement membrane of the VM tube and also promotes VM tube formation [21]. Further, targeting the abnormally secreted proteoglycan SDC1 in ovarian cancer can inhibit tumor cells from forming tubule-like structures in vitro and in vivo [203]. In addition, ECM components can further affect the differentiation direction of CSCs. These glioma stem cells can be activated by the highly expressed VEGFR-2 and VE-cadherin in the ECM laminin network to help CSCs obtain an EC-like phenotype and directly form tumor vasculature [102]. Other cellular components in ECM, such as cancer-associated fibroblasts (CAF) in melanoma, can also affect the formation of VM structures. During the formation of VM structures in melanoma, CAF malignantly overexpress the stromal cell protein CCN2 (formerly known as connective tissue growth factor, CTGF); when CAF activation is inhibited, the formation of VM structures is significantly affected [204]. Invasive VM tumor cells can support the formation of a vascular network by producing PDGF and recruiting pericytes, thus reflecting the behavior of normal blood vessels. Similar to normal blood vessels, pericytes can stabilize blood vessels lined with tumor cells and stimulate the deposition of matrix proteins to provide structural support [205]. Thus, production of ECM components in the VM network structure is an important factor that affects VM, but the specific underlying mechanism needs further research. Development of drugs targeting the “soil” of cell growth is also a good strategy.

### Drugs that may be used for clinical treatment to target vasculogenic mimicry

In tumor therapy, development of targeted drugs for angiogenesis has always been a research hotspot. However, in recent years, the clinical effects of these drugs have been unsatisfactory. Many studies have suggested that this is related to the activation of VM. In Table 1, we first summarized some drugs that are traditionally used for anti-vascular treatment but are not effective against VM or even induce VM, followed by some of the latest effective drugs for the VM generation process, and finally by drugs that target VM and are also effective against endothelial-dependent angiogenesis (Table 1).

**Table 1:** Summary of current drugs targeting endothelium-dependent angiogenesis and/or Vasculogenic mimicry

Potential drugs	Molecular target or function	Effect on EDV	Effect on VM	reference
**Angiogenesis inhibitor**				
bevacizumab	VEGF	Inhibition	No effect	[206, 207]
endostatin	Promote apoptosis of endothelial cells	Inhibition	No effect	[208, 209]
Sunitinib	RTK, VEGF-R1, VEGF-R2, VEGF-R3, PDGFR-β, KIT, FLT-3, RET	Inhibition	No effect	[210, 211]
gefitinib	EGFR	Inhibition	No effect	[212, 213]
**Vasculogenic mimicry inhibitors**				
Galunisertib (LY2157299 monohydrate)	TGF-β1, Akt, Flk	No effect	inhibition	[214]
Dequalinium (DQA) modified paclitaxel plus ligustrazine micelles	VEGF, MMP2, TGF-β1, E-cadherin	No effect	inhibition	[215]
Vinorelbine cationic liposomes modified with wheat germ	MMP-2, MMP-9,FAK\PI3K	No effect	inhibition	[216]
multifunctional targeting epirubicin liposomes	PI3K, MMP-2, MMP-9, FAK, VE-cadherin	No effect	inhibition	[217]
R 8 modified epirubicin-dihydroartemisinin liposomes	VE-cadherin/TGF-β/ MMP-2 / HIF-1	No effect	Inhibition	[218]
Tivantinib (ARQ 197)	Vnculin / RhoC	No effect	Inhibition	[219]
daunorubicin and dioscin codelivery liposomes modified with PFV	MMP-2 / VE-cadherin /TGF-β/HIF-1α	No effect	Inhibition	[220]
Niclosamide	miR-124 / STAT3	No effect	Inhibition	[221]
**Angiogenesis and Vasculogenic mimicry inhibitors**				
Axitinib	VEGFR1, VEGFR2, VEGFR3, PDGFRβ	inhibition	inhibition	[222, 223]
Cilengitide	NRP-1, v5 integrin	Inhibition	Inhibition	[224, 225]
Imatinib	PDGF,VEGF	inhibition	inhibition	[205, 226]
Trastuzumab	HER2 (VEGF, Cell cycle etc.)	Inhibition	inhibition	[227]
Entinostat	HADC (Histone deacetylase, (SERPINF1), VEGFA	inhibition	inhibition	[228, 229], [230]
Verteporfin	YAP, TEAD Ang2, MMP2, VE-cadherin, α-SMA	Inhibition	Inhibition	[231, 232]
cRGD-functionalized nanoparticles	α_v_β_3_	inhibition	inhibition	[233]

### Angiogenesis inhibitor

A classic angiogenesis inhibitor, works by binding to VEGF and blocking its activity. This has a significant effect in targeting endothelial cell-dependent angiogenesis. However, it is easy to acquire drug resistance, resulting in poor effects in their actual clinical application [234]. Bevacizumab is reported to activate the PI3K-AKT pathway through autophagy-induced ROS to promote kinase domain insertion receptor (KDR) phosphorylation in GSC, and to activate KDR/VEGFR-2 to induce stem cell transformation to endothelial cell-like phenotypes, resulting in VM [206]. Further evidence suggests that activation of the IL-8-CXCR2 pathway is responsible for development of resistance to traditional anti-angiogenic therapies in glioblastoma cells and the development of VM [235].

### Vasculogenic mimicry inhibitors

Many researchers are now exploring the VM-targeting effects and mechanisms of some drugs. Most of these drugs are generally used in the clinic as broad-spectrum anti-tumor drugs. For instance, Galunisertib, an anti-tumor TGF-β pathway kinase inhibitor, has been found to effectively inhibit the VM of tumors [214]. Some of these drugs have been obtained from clinical trials, whereas some anti-tumor drugs are modified from traditional drugs like R 8 modified epirubicin-dihydroartemisinin liposomes [218].

### Angiogenesis and vasculogenic mimicry inhibitors

Undoubtedly, an effective anti-angiogenesis strategy should inhibit both VM and endothelium-dependent blood vessels simultaneously, and the two can cooperate to exert the maximum anti-angiogenesis effect. For example, treatment with a thrombin inhibitor targeting VM, such as r-Hirudin and DTIP, has shown obvious inhibitory effects on tumor angiogenesis [212]. The latest research has focused on the use of integrin αvβ3 and self-assembly engineering to prepare nanoparticle-targeted drugs that can inhibit endothelial sprouting angiogenesis and VM with significant effects [233].

## Conclusions

This review summarizes the three key components of VM under the influence hypoxia initiating factors: CSC, EET and ECM remodeling. CSC is the “seed” in the process of VM. Under the influence of hypoxic conditions, through the EET process, VM structures are formed on the “soil” reshaped by ECM. With regard to the specific molecular mechanisms of these three components, outstanding progress has been made, but some issues still need to be resolved. Increasing evidences show that CSC is involved in the development of VM. A large part of tumor cells that constitute the VM network are derived from CSCs. During the formation of VM structures, the stemness and differentiation potential of CSCs are activated and enhanced. However, the mechanism by which CSCs are affected by transdifferentiation to endothelial-like cells is still unknown. Epithelial tumor cells have acquired endothelial cell characteristics and the function of forming a pseudovascular network. This process involves transition from epithelial characteristics to an endothelial phenotype, which should be called epithelial-endothelial transition (EET). However, in the actual situation, studies on VM have shown that the molecules involved include Twist1, Slug, and ZEB, all of which are key molecules in EMT; this has resulted in VM being referred to as EMT. Another cell phenotype that has been recognized in recent years is endothelial-mesenchymal transition (EndMT), which differs from EET. Therefore, the processes involved in tumor cell VM still need further research. It is unclear whether tumor cells are transformed into more mobile and more plastic cells through EMT (EMT is known to be an important condition for enhancing the stemness of tumor cells), and then undergo the mesenchymal-endothelial transition (the inverse of EndMT Process) to become endothelial cells. Regarding extracellular matrix remodeling, a large number of studies have shown how tumor cells produce substances that remodel ECM; further, many studies have proved that the remodeled ECM is necessary for the malignant phenotype conversion of tumor cells. However, the mechanism by which these lysed fragments affect the changes in tumor cells and the effects of other cells in the ECM on tumor cells is still unclear.

We also summarized some of the currently available drugs that target VM. Tumors showing VM are insensitive to anti-angiogenic drugs targeting endothelial cells. Moreover, some chemotherapeutic drugs can support VM by inducing the stemness of CSCs. This is also the reason why many drugs against angiogenesis have shown poor efficacy. In our summary, some drugs have shown efficacy on both VM and endothelium-dependent blood vessels in experiments. In the future, we will expand from a single target to multiple targets and screen for effective VM targeting or targeting of both endothelial cells and endothelial cells. Tumor angiogenesis (including VM and endothelial cells) is an adaptation of tumor cells to hypoxia; thus, drugs developed for the hypoxia signaling pathway seem to achieve good results. Notably, blocking VM angiogenesis can prevent tumor cells from invading along the blood vessels; further, considering the importance of EMT in tumor invasion and metastasis, as well as the similarities between VM and EMT, development of VM-targeted drugs is also of great significance to inhibit tumor invasion and metastasis.

The differentiation potential of tumor stem cells is enhanced under hypoxic conditions, and they undergo epithelial-endothelial transition to acquire endothelial-like characteristics. Simultaneously, the extracellular matrix is remodeled under anoxic conditions, and a large amount of periodic acid Schiff-positive substances is secreted to support the formation of VM tubes. Finally, tumor stem cells with endotheloid characteristics adhere to the pipe-like structures, stretch, and finally connect with blood vessels to transport red blood cells and oxygen to the tumor cells.

Hypoxia is an important regulatory factor in the CSC niche, and may act indirectly or directly by inducing the adenosine/STAT3/IL-6 pathway, MAPK/ERK pathway, Notch, Wnt, Hedgehog, Hippo signaling pathway, chemical drug induction, and so on. Transcription factors such as c-Myc, Sox-2, and Oct-4 promote the multi-directional differentiation potential of CSCs.

HIF-1α can directly promote the expression of related molecules in the VM process, including Snail, ZEB2, Twist, TGF-β3 and so on. Among these, Snail, ZEB2, and Twist are EET-related transcription factors, which can directly promote VE-cadherin, fibronectin, vitronectin, and so on, and inhibit the expression of E-cadherin to promote EET and VM. Other molecules such as TGF-β3, LOL, LEF, and others promote the expression of EET-related transcription factors by promoting cell signal transduction pathways such as TGF-β, Wnt, and PI3K. HIF-1α interacts with NICD in the cytoplasm to activate the Notch pathway, and interacts with DICER to accelerate its degradation, thus affecting the non-coding RNA pathway to regulate EET.

HIF-1α can directly regulate the expression of matrix metalloproteinases such as MMP-14, MMP-9, MMP-2, and so on. First, the classical signaling pathways, VE-cadherin/EphA2/PI3K/AKT and EphA2/FAK activate MMP14, which in turn activates MMP2. The latter two together induce Ln5 lysis and promote the occurrence of VM. In addition, cells undergo formation of actin-enriched membrane protrusions called invasive pseudopodia, which can recruit the proteases MMP-2, MMP-9 and MMP-14 to their leading edge, where they degrade the collagen and ECM basement membrane.

## Data Availability

Not application.

## Abbreviations

VM: Vasculogenic mimicry
HIF: Hypoxia inducible factor
PHD: Prolyl hydroxylase domain
CSCs: Cancer stem cells
EET: Epithelial endothelial transition
ECM: Extracellular matrix
HRE: Hypoxia response element
BHLH: Basic helix-loop-helix
VHL: Von Hippel-Lindau
AML: Human acute myeloid leukemia
SCID: Severe combined immunodeficiency
GSC: Glioblastoma stem cell-like cells
ALDH: Aldehyde dehydrogenase
TME: Tumor microenvironment
CAF: Cancer associated fibroblast
ECs: Endothelial cells
MSCs: Mesenchymal stem cells
TAMs: Tumor-associated macrophages
GSK3β: Glycogen synthase kinase
MIF: Macrophage migration inhibitory factor
PAS: Periodic acid Schiff reaction
MMP: Matrix metalloproteinases
CCN2: Connective tissue growth factor

## References

[1] Wu C (2019). Analysis of status and countermeasures of cancer incidence and mortality in China. Sci China Life Sci..

[2] Folkman J (1971). Tumor angiogenesis: therapeutic implications. N Engl J Med.

[3] Jiang X (2020). The role of microenvironment in tumor angiogenesis. J Exp Clin Cancer Res..

[4] Wei F, et al. Metabolic crosstalk in the tumor microenvironment regulates antitumor immunosuppression and immunotherapy resisitance. Cell Mol Life Sci. 2020. 10.1007/s00018-020-03581-0. 10.1007/s00018-020-03581-0 PMC11072448 32654036

[5] Viallard C, Larrivée B (2017). Tumor angiogenesis and vascular normalization: alternative therapeutic targets. Angiogenesis.

[6] Yura Y (2019). Endothelial cell-specific redox gene modulation inhibits angiogenesis but promotes B16F0 tumor growth in mice. FASEB J.

[7] Maniotis AJ (1999). Vascular channel formation by human melanoma cells in vivo and in vitro: Vasculogenic mimicry. Am J Pathol.

[8] Sun B (2017). Epithelial-to-endothelial transition and cancer stem cells: two cornerstones of vasculogenic mimicry in malignant tumors. Oncotarget.

[9] Harris AL (2002). Hypoxia--a key regulatory factor in tumour growth. Nat Rev Cancer.

[10] Fan C, et al. Emerging role of metabolic reprogramming in tumor immune evasion and immunotherapy. Sci China Life Sci. 2020. 10.1007/s11427-019-1735-4. 10.1007/s11427-019-1735-4 32815067

[11] Semenza GL (1991). Hypoxia-inducible nuclear factors bind to an enhancer element located 3′ to the human erythropoietin gene. Proc Natl Acad Sci U S A.

[12] Sun B (2007). Hypoxia influences vasculogenic mimicry channel formation and tumor invasion-related protein expression in melanoma. Cancer Lett.

[13] Zhou TJ (2016). Vasculogenic mimicry and hypoxia-inducible factor-1alpha expression in cervical squamous cell carcinoma. Genet Mol Res.

[14] Liang CT (2019). Hypoxia-inducible Factor-1: a key protein for cells adapting to changes in oxygen supply. Prog Biochem Biophys..

[15] Liu K (2015). Hypoxia induced epithelial-mesenchymal transition and vasculogenic mimicry formation by promoting Bcl-2/Twist1 cooperation. Exp Mol Pathol.

[16] Sun W (2012). Overexpression of HIF-1alpha in primary gallbladder carcinoma and its relation to vasculogenic mimicry and unfavourable prognosis. Oncol Rep.

[17] Semenza GL (2012). Hypoxia-inducible factors: mediators of cancer progression and targets for cancer therapy. Trends Pharmacol Sci.

[18] Zhang X (2019). Molecular mechanisms and anticancer therapeutic strategies in Vasculogenic mimicry. J Cancer.

[19] Seftor REB (2012). Tumor cell Vasculogenic mimicry from controversy to therapeutic promise. Am J Pathol.

[20] Wang W (2010). Vasculogenic mimicry contributes to lymph node metastasis of laryngeal squamous cell carcinoma. J Exp Clin Cancer Res.

[21] Hao XS (2003). Correlation between the expression of collgen IV, VEGF and vasculogenic mimicry. Zhonghua Zhongliu Zazhi.

[22] Xiang T (2018). Vasculogenic mimicry formation in EBV-associated epithelial malignancies. Nat Commun.

[23] Chen Y-S, Chen Z-P (2014). Vasculogenic mimicry: a novel target for glioma therapy. Chin J Cancer.

[24] Valdivia A (2019). Fact or fiction, it is time for a verdict on Vasculogenic mimicry?. Front Oncol.

[25] El Hallani S (2010). A new alternative mechanism in glioblastoma vascularization: tubular vasculogenic mimicry. Brain.

[26] Yang JP (2016). Tumor vasculogenic mimicry predicts poor prognosis in cancer patients: a meta-analysis. Angiogenesis.

[27] Liu Q (2016). The relationship between vasculogenic mimicry and epithelial-mesenchymal transitions. J Cell Mol Med.

[28] Hendrix MJ (2002). Transendothelial function of human metastatic melanoma cells: role of the microenvironment in cell-fate determination. Cancer Res.

[29] Yi M (2020). TP63 links chromatin remodeling and enhancer reprogramming to epidermal differentiation and squamous cell carcinoma development. Cell Mol Life Sci..

[30] Mani SA (2008). The epithelial-mesenchymal transition generates cells with properties of stem cells. Cell.

[31] Fang D (2005). A tumorigenic subpopulation with stem cell properties in melanomas. Cancer Res.

[32] Ricci-Vitiani L (2010). Tumour vascularization via endothelial differentiation of glioblastoma stem-like cells. Nature.

[33] Wang R (2010). Glioblastoma stem-like cells give rise to tumour endothelium. Nature.

[34] Sun H (2019). Cancer stem-like cells directly participate in vasculogenic mimicry channels in triple-negative breast cancer. Cancer Biol Med.

[35] Lai CY, Schwartz BE, Hsu MY (2012). CD133+ melanoma subpopulations contribute to perivascular niche morphogenesis and tumorigenicity through vasculogenic mimicry. Cancer Res.

[36] Boesch M (2017). Harnessing the DNA dye-triggered side population phenotype to detect and purify Cancer stem cells from biological samples. J Vis Exp.

[37] Leung EL (2010). Non-small cell lung cancer cells expressing CD44 are enriched for stem cell-like properties. PLoS One.

[38] Toledo-Guzman ME (2019). ALDH as a stem cell marker in solid tumors. Curr Stem Cell Res Ther.

[39] Hendrix MJ (2003). Remodeling of the microenvironment by aggressive melanoma tumor cells. Ann N Y Acad Sci.

[40] Pistollato F (2010). Intratumoral hypoxic gradient drives stem cells distribution and MGMT expression in glioblastoma. Stem Cells.

[41] Mo Y (2019). The role of Wnt signaling pathway in tumor metabolic reprogramming. J Cancer.

[42] Cui CP (2017). SENP1 promotes hypoxia-induced cancer stemness by HIF-1alpha deSUMOylation and SENP1/HIF-1alpha positive feedback loop. Gut.

[43] Bhuria V (2019). Hypoxia induced sonic hedgehog signaling regulates cancer stemness, epithelial-to-mesenchymal transition and invasion in cholangiocarcinoma. Exp Cell Res.

[44] Vadde R (2017). Role of hypoxia-inducible factors (HIF) in the maintenance of stemness and malignancy of colorectal cancer. Crit Rev Oncol Hematol.

[45] Jacobsson H (2019). Hypoxia-induced secretion stimulates breast cancer stem cell regulatory signalling pathways. Mol Oncol.

[46] Ezashi T, Das P, Roberts RM (2005). Low O2 tensions and the prevention of differentiation of hES cells. Proc Natl Acad Sci U S A.

[47] Iida H (2012). Hypoxia induces CD133 expression in human lung cancer cells by up-regulation of OCT3/4 and SOX2. Int J Oncol.

[48] Zhang D (2014). Twist1 expression induced by sunitinib accelerates tumor cell vasculogenic mimicry by increasing the population of CD133+ cells in triple-negative breast cancer. Mol Cancer.

[49] Won C (2015). Signal transducer and activator of transcription 3-mediated CD133 up-regulation contributes to promotion of hepatocellular carcinoma. Hepatology.

[50] Pietras A (2014). Osteopontin-CD44 signaling in the glioma perivascular niche enhances cancer stem cell phenotypes and promotes aggressive tumor growth. Cell Stem Cell.

[51] Fan YL (2013). A new perspective of vasculogenic mimicry: EMT and cancer stem cells (review). Oncol Lett.

[52] Xing P (2018). ALDH1 expression and Vasculogenic mimicry are positively associated with poor prognosis in patients with breast Cancer. Cell Physiol Biochem.

[53] Wang HF (2019). Hypoxia promotes vasculogenic mimicry formation by vascular endothelial growth factor a mediating epithelial-mesenchymal transition in salivary adenoid cystic carcinoma. Cell Prolif.

[54] Bao Z, Cheng Z, Chai D (2018). The expressions of CD133, ALDH1, and vasculogenic mimicry in osteosarcoma and their clinical significance. Int J Clin Exp Pathol.

[55] Ding YP (2014). Autophagy promotes the survival and development of tumors by participating in the formation of vasculogenic mimicry. Oncol Rep.

[56] Mohiuddin IS, Wei SJ, Kang MH (1866). Role of OCT4 in cancer stem-like cells and chemotherapy resistance. Biochim Biophys Acta Mol basis Dis.

[57] Das B (2019). MYC regulates the HIF2alpha Stemness pathway via Nanog and Sox2 to maintain self-renewal in Cancer stem cells versus non-stem Cancer cells. Cancer Res.

[58] Jeter CR (2015). Concise review: NANOG in Cancer stem cells and tumor development: an update and outstanding questions. Stem Cells.

[59] Zhang C (2016). Hypoxia induces the breast cancer stem cell phenotype by HIF-dependent and ALKBH5-mediated m (6) A-demethylation of NANOG mRNA. Proc Natl Acad Sci U S A.

[60] McCord AM (2009). Physiologic oxygen concentration enhances the stem-like properties of CD133+ human glioblastoma cells in vitro. Mol Cancer Res.

[61] Qiang L (2012). HIF-1alpha is critical for hypoxia-mediated maintenance of glioblastoma stem cells by activating notch signaling pathway. Cell Death Differ.

[62] Schroeter EH, Kisslinger JA, Kopan R (1998). Notch-1 signalling requires ligand-induced proteolytic release of intracellular domain. Nature.

[63] Gustafsson MV (2005). Hypoxia requires notch signaling to maintain the undifferentiated cell state. Dev Cell.

[64] Li Y (2018). HIF-1 alpha is critical for the activation of notch signaling in neurogenesis during acute epilepsy. Neuroscience.

[65] Seo EJ (2016). Hypoxia-NOTCH1-SOX2 signaling is important for maintaining cancer stem cells in ovarian cancer. Oncotarget.

[66] Man J (2018). Hypoxic induction of Vasorin regulates Notch1 turnover to maintain Glioma stem-like cells. Cell Stem Cell.

[67] Hu YY (2014). Hif-1alpha and Hif-2alpha differentially regulate notch signaling through competitive interaction with the intracellular domain of notch receptors in glioma stem cells. Cancer Lett.

[68] Venkatesh V (2018). Targeting notch signalling pathway of cancer stem cells. Stem Cell Investig.

[69] Pai VC (2019). ASPM promotes prostate cancer stemness and progression by augmenting Wnt-Dvl-3-beta-catenin signaling. Oncogene.

[70] Lee SH (2014). Wnt/beta-catenin signalling maintains self-renewal and tumourigenicity of head and neck squamous cell carcinoma stem-like cells by activating Oct4. J Pathol.

[71] Mei Y (2018). RIF1 promotes tumor growth and cancer stem cell-like traits in NSCLC by protein phosphatase 1-mediated activation of Wnt/beta-catenin signaling. Cell Death Dis.

[72] Reya T, Clevers H (2005). Wnt signalling in stem cells and cancer. Nature.

[73] Liu HL (2015). Hypoxia-inducible factor-1alpha and Wnt/beta-catenin signaling pathways promote the invasion of hypoxic gastric cancer cells. Mol Med Rep.

[74] Xu W (2017). Hypoxia activates Wnt/beta-catenin signaling by regulating the expression of BCL9 in human hepatocellular carcinoma. Sci Rep.

[75] Zhao J (2020). Single cell RNA-seq reveals the landscape of tumor and infiltrating immune cells in nasopharyngeal carcinoma. Cancer Lett..

[76] Kaidi A, Williams AC, Paraskeva C (2007). Interaction between beta-catenin and HIF-1 promotes cellular adaptation to hypoxia. Nat Cell Biol.

[77] Yan Y (2018). HIF-2alpha promotes conversion to a stem cell phenotype and induces chemoresistance in breast cancer cells by activating Wnt and notch pathways. J Exp Clin Cancer Res.

[78] Lin X (2017). C-myc overexpression drives melanoma metastasis by promoting vasculogenic mimicry via c-myc/snail/Bax signaling. J Mol Med (Berl).

[79] Liu T (2014). OCT4 expression and vasculogenic mimicry formation positively correlate with poor prognosis in human breast cancer. Int J Mol Sci.

[80] Gordan JD (2007). HIF-2 alpha promotes hypoxic cell proliferation by enhancing c-Myc transcriptional activity. Cancer Cell.

[81] Lu JT (2012). Hedgehog signaling pathway mediates invasion and metastasis of hepatocellular carcinoma via ERK pathway. Acta Pharmacol Sin.

[82] Wang X (2012). Sonic hedgehog regulates Bmi1 in human medulloblastoma brain tumor-initiating cells. Oncogene.

[83] Liu Z (2017). Hypoxia accelerates aggressiveness of hepatocellular carcinoma cells involving oxidative stress, epithelial-Mesenchymal transition and non-canonical hedgehog signaling. Cell Physiol Biochem.

[84] Li Z (2015). The hippo transducer TAZ promotes epithelial to mesenchymal transition and cancer stem cell maintenance in oral cancer. Mol Oncol.

[85] Xiang L (2014). Hypoxia-inducible factor 1 mediates TAZ expression and nuclear localization to induce the breast cancer stem cell phenotype. Oncotarget.

[86] Ma B (2015). Hypoxia regulates hippo signalling through the SIAH2 ubiquitin E3 ligase. Nat Cell Biol.

[87] Xiang L (2015). HIF-1alpha and TAZ serve as reciprocal co-activators in human breast cancer cells. Oncotarget.

[88] Bora-Singhal N (2015). YAP1 regulates OCT4 activity and SOX2 expression to facilitate self-renewal and vascular mimicry of stem-like cells. Stem Cells.

[89] Samanta D (2014). Hypoxia-inducible factors are required for chemotherapy resistance of breast cancer stem cells. Proc Natl Acad Sci U S A.

[90] Lu H (2015). Chemotherapy triggers HIF-1-dependent glutathione synthesis and copper chelation that induces the breast cancer stem cell phenotype. Proc Natl Acad Sci U S A.

[91] Lu H (2018). Reciprocal regulation of DUSP9 and DUSP16 expression by HIF1 controls ERK and p38 MAP kinase activity and mediates chemotherapy-induced breast Cancer stem cell enrichment. Cancer Res.

[92] Lu H (2017). Chemotherapy-induced Ca (2+) release stimulates breast Cancer stem cell enrichment. Cell Rep.

[93] Johnson DE, O'Keefe RA, Grandis JR (2018). Targeting the IL-6/JAK/STAT3 signalling axis in cancer. Nat Rev Clin Oncol.

[94] Lan J (2018). Hypoxia-inducible factor 1-dependent expression of adenosine receptor 2B promotes breast cancer stem cell enrichment. Proc Natl Acad Sci U S A.

[95] Pierobon M (2017). Enrichment of PI3K-AKT-mTOR pathway activation in hepatic metastases from breast Cancer. Clin Cancer Res.

[96] Bai J (2020). HIF-2alpha regulates CD44 to promote cancer stem cell activation in triple-negative breast cancer via PI3K/AKT/mTOR signaling. World J Stem Cells.

[97] Zhao H, Gu X-M (2008). Study on vasculogenic mimicry in malignant esophageal stromal tumors. World J Gastroenterol.

[98] Mercurio AM (2019). VEGF/Neuropilin Signaling in Cancer Stem Cells. Int J Mol Sci.

[99] Beck B (2011). A vascular niche and a VEGF-Nrp1 loop regulate the initiation and stemness of skin tumours. Nature.

[100] Goel HL, Mercurio AM (2013). VEGF targets the tumour cell. Nat Rev Cancer.

[101] Jin K (2019). Long non-coding RNA PVT1 interacts with MYC and its downstream molecules to synergistically promote tumorigenesis. Cell Mol Life Sci..

[102] Yao X (2013). Vascular endothelial growth factor receptor 2 (VEGFR-2) plays a key role in vasculogenic mimicry formation, neovascularization and tumor initiation by Glioma stem-like cells. PLoS One.

[103] Deng X (2020). LncRNA LINC00472 regulates cell stiffness and inhibits the migration and invasion of lung adenocarcinoma by binding to YBX1. Cell Death Dis..

[104] Xu X (2019). VEGF induce Vasculogenic mimicry of Choroidal melanoma through the PI3k signal pathway. Biomed Res Int.

[105] Hapke RY, Haake SM (2020). Hypoxia-induced epithelial to mesenchymal transition in cancer. Cancer Lett.

[106] Tang L, et al. circSETD3 regulates MAPRE1 through miR-615-5p and miR-1538 sponges to promote migration and invasion in nasopharyngeal carcinoma. Oncogene. 2020. 10.1038/s41388-020-01531-5. 10.1038/s41388-020-01531-5 33122825

[107] Wu Y (2020). EBV-miR-BART12 accelerates migration and invasion in EBV-associated cancer cells by targeting tubulin polymerization-promoting protein 1. FASEB J..

[108] Hugo HJ (2011). Defining the E-cadherin repressor Interactome in epithelial-Mesenchymal transition: the PMC42 model as a case study. Cells Tissues Organs.

[109] Delgado-Bellido D (2017). Vasculogenic mimicry signaling revisited: focus on non-vascular VE-cadherin. Mol Cancer.

[110] Sun T (2010). Expression and functional significance of Twist1 in hepatocellular carcinoma: its role in vasculogenic mimicry. Hepatology.

[111] Zhu P (2010). The proliferation, apoptosis, invasion of endothelial-like epithelial ovarian cancer cells induced by hypoxia. J Exp Clin Cancer Res.

[112] Li W (2016). Hypoxia-induced vasculogenic mimicry formation in human colorectal cancer cells: involvement of HIF-1a, Claudin-4, and E-cadherin and Vimentin. Sci Rep.

[113] Lin H (2019). Vimentin Overexpressions induced by cell hypoxia promote Vasculogenic mimicry by renal cell carcinoma cells. Biomed Res Int.

[114] Liu K (2015). Hypoxia promotes vasculogenic mimicry formation by the Twist1-Bmi1 connection in hepatocellular carcinoma. Int J Mol Med.

[115] De Francesco EM, Maggiolini M, Musti AM (2018). Crosstalk between Notch, HIF-1alpha and GPER in Breast Cancer EMT. Int J Mol Sci.

[116] Zhang Q (2013). Wnt/beta-catenin signaling enhances hypoxia-induced epithelial-mesenchymal transition in hepatocellular carcinoma via crosstalk with hif-1alpha signaling. Carcinogenesis.

[117] Tang T (2020). LncRNA AATBC regulates Pinin to promote metastasis in nasopharyngeal carcinoma. Mol Oncol..

[118] Yang MH (2008). Direct regulation of TWIST by HIF-1alpha promotes metastasis. Nat Cell Biol.

[119] Zhu GH (2013). Hypoxia-induced snail expression through transcriptional regulation by HIF-1 alpha in pancreatic cancer cells. Dig Dis Sci.

[120] Nakuluri K (2019). Hypoxia induces ZEB2 in podocytes: implications in the pathogenesis of proteinuria. J Cell Physiol.

[121] Yang J (2004). Twist, a master regulator of morphogenesis, plays an essential role in tumor metastasis. Cell.

[122] Murre C (1989). Interactions between heterologous helix-loop-helix proteins generate complexes that bind specifically to a common DNA sequence. Cell.

[123] Onder TT (2008). Loss of E-cadherin promotes metastasis via multiple downstream transcriptional pathways. Cancer Res.

[124] Yang J (2017). HIF-2α promotes the formation of vasculogenic mimicry in pancreatic cancer by regulating the binding of Twist1 to the VE-cadherin promoter. Oncotarget.

[125] Tan R (2017). Expression and significance of Twist, estrogen receptor, and E-cadherin in human breast cancer cells and tissues. J Cancer Res Ther.

[126] Yang J (2016). HIF-2alpha promotes epithelial-mesenchymal transition through regulating Twist2 binding to the promoter of E-cadherin in pancreatic cancer. J Exp Clin Cancer Res.

[127] Meng J (2018). Twist1 regulates Vimentin through Cul2 circular RNA to promote EMT in hepatocellular carcinoma. Cancer Res.

[128] Li J (2014). Endothelial TWIST1 promotes pathological ocular angiogenesis. Invest Ophthalmol Vis Sci.

[129] Yang M-H (2010). Bmi1 is essential in Twist1-induced epithelial-mesenchymal transition. Nat Cell Biol.

[130] Zhao N (2012). Hypoxia-induced vasculogenic mimicry formation via VE-cadherin regulation by Bcl-2. Med Oncol.

[131] Duan Y (2017). Hypoxia induced Bcl-2/Twist1 complex promotes tumor cell invasion in oral squamous cell carcinoma. Oncotarget.

[132] Wang L (2015). Metastasis-associated in colon cancer-1 promotes vasculogenic mimicry in gastric cancer by upregulating TWIST1/2. Oncotarget.

[133] Cao J (2018). Twist promotes tumor metastasis in basal-like breast cancer by transcriptionally upregulating ROR1. Theranostics.

[134] Kaufhold S, Bonavida B (2014). Central role of Snail1 in the regulation of EMT and resistance in cancer: a target for therapeutic intervention. J Exp Clin Cancer Res.

[135] Sun D (2013). Slug promoted vasculogenic mimicry in hepatocellular carcinoma. J Cell Mol Med.

[136] Batlle E (2000). The transcription factor snail is a repressor of E-cadherin gene expression in epithelial tumour cells. Nat Cell Biol.

[137] Evans AJ (2007). VHL promotes E2 box-dependent E-cadherin transcription by HIF-mediated regulation of SIP1 and snail. Mol Cell Biol.

[138] Zhou BP (2004). Dual regulation of Snail by GSK-3 beta-mediated phosphorylation in control of epithelial-mesenchymal transition. Nat Cell Biol.

[139] Smith-Mungo LI, Kagan HM (1998). Lysyl oxidase: properties, regulation and multiple functions in biology. Matrix Biol.

[140] Shao B (2019). LOXL2 promotes vasculogenic mimicry and tumour aggressiveness in hepatocellular carcinoma. J Cell Mol Med.

[141] Wang M (2017). HIF-1α promoted vasculogenic mimicry formation in hepatocellular carcinoma through LOXL2 up-regulation in hypoxic tumor microenvironment. J Exp Clin Cancer Res.

[142] Peinado H, Portillo F, Cano A (2005). Switching on-off snail - LOXL2 versus GSK3 beta. Cell Cycle.

[143] Fan Z (2020). LOXL2 upregulates hypoxia-inducible factor-1 alpha signaling through snail-FBP1 axis in hepatocellular carcinoma cells. Oncol Rep.

[144] Shi X (2018). Hypotoxic copper complexes with potent anti-metastatic and anti-angiogenic activities against cancer cells. Dalton Trans.

[145] Yang Z (2015). ZEB2 promotes vasculogenic mimicry by TGF-β1 induced epithelial-to-mesenchymal transition in hepatocellular carcinoma. Exp Mol Pathol.

[146] Yu L (2014). Tumor necrosis factor α induces epithelial-mesenchymal transition and promotes metastasis via NF-κB signaling pathway-mediated TWIST expression in hypopharyngeal cancer. Oncol Rep.

[147] Pires BR (2017). NF-kappaB Is Involved in the Regulation of EMT Genes in Breast Cancer Cells. PLoS One.

[148] Šošić D (2003). Twist regulates cytokine gene expression through a negative feedback loop that represses NF-kappaB activity. Cell.

[149] Arsura M (2000). Role of the IkappaB kinase complex in oncogenic Ras- and Raf-mediated transformation of rat liver epithelial cells. Mol Cell Biol.

[150] Zhang J, Tian XJ, Xing J (2016). Signal transduction pathways of EMT Induced by TGF-β, SHH, and WNT and their Crosstalks. J Clin Med.

[151] Howe LR (2003). Twist is up-regulated in response to Wnt1 and inhibits mouse mammary cell differentiation. Cancer Res.

[152] Qi L (2015). Wnt3a promotes the Vasculogenic mimicry formation of Colon Cancer via Wnt/beta-catenin signaling. Int J Mol Sci.

[153] Vartanian A (2013). The involvement of notch signaling in melanoma vasculogenic mimicry. Clin Exp Med.

[154] Hunkapiller NM (2011). A role for notch signaling in trophoblast endovascular invasion and in the pathogenesis of pre-eclampsia. Development.

[155] Wang D (2020). Epstein-Barr virus-encoded miR-BART6-3p inhibits cancer cell proliferation through the LOC553103-STMN1 axis. FASEB J..

[156] de Larco JE, Todaro GJ (1978). Growth factors from murine sarcoma virus-transformed cells. Proc Natl Acad Sci U S A.

[157] Derynck R (1996). Nomenclature: vertebrate mediators of TGFbeta family signals. Cell.

[158] Whitman M (1998). Smads and early developmental signaling by the TGFbeta superfamily. Genes Dev.

[159] Feng XH, Derynck R (2005). Specificity and versatility in TGF-beta signaling through Smads. Annu Rev Cell Dev Biol.

[160] Hao Y, Baker D, ten Dijke P (2019). TGF--Mediated Epithelial-Mesenchymal Transition and Cancer Metastasis. Int J Mol Sci.

[161] Cho HJ (2007). Snail is required for transforming growth factor-beta-induced epithelial-mesenchymal transition by activating PI3 kinase/Akt signal pathway. Biochem Biophys Res Commun.

[162] Bakin AV (2000). Phosphatidylinositol 3-kinase function is required for transforming growth factor beta-mediated epithelial to mesenchymal transition and cell migration. J Biol Chem.

[163] Zhang J, Tian X-J, Xing J (2016). Signal Transduction Pathways of EMT Induced by TGF-, SHH, and WNT and Their Crosstalks. J Clin Med.

[164] Secker GA (2008). TGF beta stimulated re-epithelialisation is regulated by CTGF and Ras/MEK/ERK signalling. Exp Cell Res.

[165] Xu J, Lamouille S, Derynck R (2009). TGF-beta-induced epithelial to mesenchymal transition. Cell Res.

[166] Wang J (2015). Hypoxia inducible factor-1-dependent up-regulation of BMP4 mediates hypoxia-induced increase of TRPC expression in PASMCs. Cardiovasc Res.

[167] Li X (2020). Function of BMP4 in the formation of Vasculogenic mimicry in hepatocellular carcinoma. J Cancer.

[168] Frey P (2020). Canonical BMP Signaling Executes Epithelial-Mesenchymal Transition Downstream of SNAIL1. Cancers.

[169] Gong W (2016). Nodal signaling promotes vasculogenic mimicry formation in breast cancer via the Smad2/3 pathway. Oncotarget.

[170] Caniggia I (2000). Hypoxia-inducible factor-1 mediates the biological effects of oxygen on human trophoblast differentiation through TGFbeta (3). J Clin Invest.

[171] Copple BL (2010). Hypoxia stimulates hepatocyte epithelial to mesenchymal transition by hypoxia-inducible factor and transforming growth factor-beta-dependent mechanisms. Liver Int.

[172] Han WQ (2013). Hypoxia-inducible factor prolyl-hydroxylase-2 mediates transforming growth factor beta 1-induced epithelial-mesenchymal transition in renal tubular cells. Biochim Biophys Acta.

[173] Lai HH (2018). HIF-1α promotes autophagic proteolysis of Dicer and enhances tumor metastasis. J Clin Invest.

[174] Richard V, Kindt N, Saussez S (2015). Macrophage migration inhibitory factor involvement in breast cancer (review). Int J Oncol.

[175] Sun L (2008). Transforming growth factor-beta 1 promotes matrix metalloproteinase-9-mediated ora cancer invasion through snail expression. Mol Cancer Res.

[176] Miyoshi A (2004). Snail and SIP1 increase cancer invasion by upregulating MMP family in hepatocellular carcinoma cells. Br J Cancer.

[177] Qie S (2009). Correlation between expressions of matrix metalloproteinase-2 & 9 and vasculogenic mimicry in gastrointestinal stromal tumors. Zhonghua Yi Xue Za Zhi.

[178] Bergers G (2000). Matrix metalloproteinase-9 triggers the angiogenic switch during carcinogenesis. Nat Cell Biol.

[179] Yu Q, Stamenkovic I (2000). Cell surface-localized matrix metalloproteinase-9 proteolytically activates TGF-beta and promotes tumor invasion and angiogenesis. Genes Dev.

[180] Nistico P, Bissell MJ, Radisky DC (2012). Epithelial-mesenchymal transition: general principles and pathological relevance with special emphasis on the role of matrix metalloproteinases. Cold Spring Harb Perspect Biol.

[181] Dahl KDC (2008). Matrix metalloproteinase 9 is a mediator of epidermal growth factor-dependent E-cadherin loss in ovarian carcinoma cells. Cancer Res.

[182] Gall TMH, Frampton AE (2013). Gene of the month: E-cadherin (CDH1). J Clin Pathol.

[183] Duxbury MS (2004). EphA2: a determinant of malignant cellular behavior and a potential therapeutic target in pancreatic adenocarcinoma. Oncogene.

[184] Hess AR (2003). Phosphoinositide 3-kinase regulates membrane type 1-matrix metalloproteinase (MMP) and MMP-2 activity during melanoma cell vasculogenic mimicry. Cancer Res.

[185] Ge H, Luo H (2018). Overview of advances in vasculogenic mimicry - a potential target for tumor therapy. Cancer Manag Res.

[186] Williamson SC (2016). Vasculogenic mimicry in small cell lung cancer. Nat Commun.

[187] Le Bras A (2007). HIF-2alpha specifically activates the VE-cadherin promoter independently of hypoxia and in synergy with ETS-1 through two essential ETS-binding sites. Oncogene.

[188] Kurose H (2019). Elevated expression of EPHA2 is associated with poor prognosis after radical prostatectomy in prostate Cancer. Anticancer Res.

[189] Kim HS (2019). Morphological characteristics of vasculogenic mimicry and its correlation with EphA2 expression in gastric adenocarcinoma. Sci Rep.

[190] Hess AR (2001). Molecular regulation of tumor cell vasculogenic mimicry by tyrosine phosphorylation: role of epithelial cell kinase (Eck/EphA2). Cancer Res.

[191] Pandey A (1994). Activation of the Eck receptor protein tyrosine kinase stimulates phosphatidylinositol 3-kinase activity. J Biol Chem.

[192] Tang Y (2016). 14–3-3 beta Promotes Migration and Invasion of Human Hepatocellular Carcinoma Cells by Modulating Expression of MMP2 and MMP9 through PI3K/Akt/NF-kappa B Pathway. PLoS One.

[193] Lissitzky JC (2009). Cyclic AMP signaling as a mediator of vasculogenic mimicry in aggressive human melanoma cells in vitro. Cancer Res.

[194] Qi H (2014). Wnt5a promotes vasculogenic mimicry and epithelial-mesenchymal transition via protein kinase Calpha in epithelial ovarian cancer. Oncol Rep.

[195] Lu XS (2013). Contribution of the PI3K/MMPs/Ln-5γ2 and EphA2/FAK/Paxillin signaling pathways to tumor growth and vasculogenic mimicry of gallbladder carcinomas. Int J Oncol.

[196] Hess AR, Hendrix MJ (2006). Focal adhesion kinase signaling and the aggressive melanoma phenotype. Cell Cycle.

[197] Seftor RE (2001). Cooperative interactions of laminin 5 gamma2 chain, matrix metalloproteinase-2, and membrane type-1-matrix/metalloproteinase are required for mimicry of embryonic vasculogenesis by aggressive melanoma. Cancer Res.

[198] Yousif LF, Di Russo J, Sorokin L (2013). Laminin isoforms in endothelial and perivascular basement membranes. Cell Adhes Migr.

[199] Eckert MA (2011). Twist1-induced Invadopodia formation promotes tumor metastasis. Cancer Cell.

[200] Stylli SS, Kaye AH, Lock P (2008). Invadopodia: at the cutting edge of tumour invasion. J Clin Neurosci.

[201] Song D-G (2017). TM4SF5 promotes metastatic behavior of cells in 3D extracellular matrix gels by reducing dependency on environmental cues. Oncotarget.

[202] Hendrix MJ, Nilsen M, Hamilton ZW, Keshet E (2003). Remodeling of the microenvironment by aggressive melanoma tumor cells in Tissue Remodeling.

[203] Orecchia P (2019). L19-IL2 Immunocytokine in Combination with the Anti-Syndecan-1 46F2SIP Antibody Format: A New Targeted Treatment Approach in an Ovarian Carcinoma Model. Cancers (Basel)..

[204] Hutchenreuther J (2018). Activation of cancer-associated fibroblasts is required for tumor neovascularization in a murine model of melanoma. Matrix Biol.

[205] Thijssen VL (2018). Targeting PDGF-mediated recruitment of pericytes blocks vascular mimicry and tumor growth. J Pathol.

[206] Wu H-B (2017). Autophagy-induced KDR/VEGFR-2 activation promotes the formation of vasculogenic mimicry by glioma stem cells. Autophagy.

[207] Vredenburgh JJ (2007). Bevacizumab plus irinotecan in recurrent glioblastoma multiforme. J Clin Oncol.

[208] O'Reilly MS (1997). Endostatin: an endogenous inhibitor of angiogenesis and tumor growth. Cell.

[209] Qu B (2018). MIG7 is involved in vasculogenic mimicry formation rendering invasion and metastasis in hepatocellular carcinoma. Oncol Rep.

[210] Motzer RJ (2007). Sunitinib versus interferon alfa in metastatic renal-cell carcinoma. N Engl J Med.

[211] Sun H (2017). Anti-angiogenic treatment promotes triple-negative breast cancer invasion via vasculogenic mimicry. Cancer Biol Ther.

[212] Zhao B (2020). Thrombin is a therapeutic target for non-small-cell lung cancer to inhibit vasculogenic mimicry formation. Signal Transduct Target Ther.

[213] Chen S (2020). EGFR-PKM2 signaling promotes the metastatic potential of nasopharyngeal carcinoma through induction of FOSL1 and ANTXR2. Carcinogenesis..

[214] Zhang C (2016). Galunisertib inhibits glioma vasculogenic mimicry formation induced by astrocytes. Sci Rep.

[215] Xie HJ (2019). Inhibiting tumour metastasis by DQA modified paclitaxel plus ligustrazine micelles in treatment of non-small-cell lung cancer. Artif Cells Nanomed Biotechnol.

[216] Xiao Y (2018). Vinorelbine cationic liposomes modified with wheat germ agglutinin for inhibiting tumor metastasis in treatment of brain glioma. Artif Cells Nanomed Biotechnol.

[217] Song XL (2017). Application of multifunctional targeting epirubicin liposomes in the treatment of non-small-cell lung cancer. Int J Nanomedicine.

[218] Liu JJ (2019). Development of R8 modified epirubicin-dihydroartemisinin liposomes for treatment of non-small-cell lung cancer. Artif Cells Nanomed Biotechnol.

[219] Kumar SR (2019). Molecular targets for tivantinib (ARQ 197) and vasculogenic mimicry in human melanoma cells. Eur J Pharmacol.

[220] Wang Y (2019). Inhibition of tumor metastasis by targeted daunorubicin and dioscin codelivery liposomes modified with PFV for the treatment of non-small-cell lung cancer. Int J Nanomedicine.

[221] Li X (2018). Niclosamide acts as a new inhibitor of vasculogenic mimicry in oral cancer through upregulation of miR-124 and downregulation of STAT3. Oncol Rep.

[222] Tortorici MA (2014). Pharmacokinetics of single-agent axitinib across multiple solid tumor types. Cancer Chemother Pharmacol.

[223] Bedal KB (2015). The NC11 domain of human collagen XVI induces vasculogenic mimicry in oral squamous cell carcinoma cells. Carcinogenesis.

[224] Ruffini F (2015). Cilengitide downmodulates invasiveness and vasculogenic mimicry of neuropilin 1 expressing melanoma cells through the inhibition of alphavbeta5 integrin. Int J Cancer.

[225] Reardon DA (2008). Cilengitide: an integrin-targeting arginine-glycine-aspartic acid peptide with promising activity for glioblastoma multiforme. Expert Opin Investig Drugs.

[226] Cohen NA (2013). Angiogenesis inhibition augments the effect of imatinib in gastrointestinal stromal tumor. J Am Coll Surg.

[227] Hori A (2019). Vasculogenic mimicry is associated with trastuzumab resistance of HER2-positive breast cancer. Breast Cancer Res.

[228] Srivastava RK, Kurzrock R, Shankar S (2010). MS-275 sensitizes TRAIL-resistant breast Cancer cells, inhibits angiogenesis and metastasis, and reverses epithelial-Mesenchymal transition in vivo. Mol Cancer Ther.

[229] Maiti A (2019). Class I histone deacetylase inhibitor suppresses vasculogenic mimicry by enhancing the expression of tumor suppressor and anti-angiogenesis genes in aggressive human TNBC cells. Int J Oncol.

[230] Pastorino O (2019). Histone deacetylase inhibitors impair vasculogenic mimicry from glioblastoma cells. Cancers.

[231] Wei H (2017). Verteporfin suppresses cell survival, angiogenesis and vasculogenic mimicry of pancreatic ductal adenocarcinoma via disrupting the YAP-TEAD complex. Cancer Sci.

[232] Chen B (2008). Disparity between prostate tumor interior versus peripheral vasculature in response to verteporfin-mediated vascular-targeting therapy. Int J Cancer.

[233] Wang Y (2019). cRGD-functionalized nanoparticles for combination therapy of anti-endothelium dependent vessels and anti-vasculogenic mimicry to inhibit the proliferation of ovarian cancer. Acta Biomater.

[234] Lacal PM, Graziani G (2018). Therapeutic implication of vascular endothelial growth factor receptor-1 (VEGFR-1) targeting in cancer cells and tumor microenvironment by competitive and non-competitive inhibitors. Pharmacol Res.

[235] Angara K (2018). CXCR2-expressing tumor cells drive vascular mimicry in Antiangiogenic therapy-resistant Glioblastoma. Neoplasia.

